# Novel brown adipose tissue candidate genes predicted by the human gene connectome

**DOI:** 10.1038/s41598-022-11317-2

**Published:** 2022-05-09

**Authors:** Diego F. Salazar-Tortosa, David Enard, Yuval Itan, Jonatan R. Ruiz

**Affiliations:** 1 PROFITH ‘PROmoting FITness and Health Through Physical Activity’ Research Group, Sport and Health University Research Institute (iMUDS), University of Granada, Granada, Spain; 2 Department of Ecology and Evolutionary Biology, University of Arizona, Tucson, AZ USA; 3 The Charles Bronfman Institute of Personalized Medicine, Icahn School of Medicine at Mount Sinai, New York, NY USA; 4 Department of Genetics and Genomic Sciences, Icahn School of Medicine at Mount Sinai, New York, NY USA; 5 Department of Physical Education and Sport, Faculty of Sport Sciences, University of Granada, Granada, Spain; 6 Instituto de Investigación Biosanitaria, Ibs.Granada, Granada, Spain

**Keywords:** Computational biology and bioinformatics, Genetics, Metabolism

## Abstract

Brown adipose tissue (BAT) is a promising therapeutic target against obesity. Therefore, research on the genetic architecture of BAT could be key for the development of successful therapies against this complex phenotype. Hypothesis-driven candidate gene association studies are useful for studying genetic determinants of complex traits, but they are dependent upon the previous knowledge to select candidate genes. Here, we predicted 107 novel-BAT candidate genes in silico using the uncoupling protein one (UCP1) as the hallmark of BAT activity. We first identified the top 1% of human genes predicted by the human gene connectome to be biologically closest to the UCP1, estimating 167 additional pathway genes (BAT connectome). We validated this prediction by showing that 60 genes already associated with BAT were included in the connectome and they were biologically closer to each other than expected by chance (p < 2.2 × 10^−16^). The rest of genes (107) are potential candidates for BAT, being also closer to known BAT genes and more expressed in BAT biopsies than expected by chance (p < 2.2 × 10^−16^; p = 4.39 × 10^–02^). The resulting new list of predicted human BAT genes should be useful for the discovery of novel BAT genes and metabolic pathways.

## Introduction

Brown adipose tissue (BAT) is a possible therapeutic target against obesity and related disorders due to its ability to oxidise glucose and lipids and to dissipate energy as heat^1–3^. Therefore, research on the genetic architecture of BAT could be key for the development of successful therapies against obesity. Hypothesis-driven candidate gene association studies are a useful approach to study the genetic determinants of complex traits^4^. However, this approach depends on the previous knowledge to formulate and test association hypothesis. Here, we used the human gene connectome (HGC) to predict novel-candidate genes for BAT. The HGC is a set of biological distances and routes of interaction predicted in silico by a shortest distance algorithm applied to the full human genome network, which is conceptually similar to GPS navigation^5^. Therefore, these biological distances are based on information about protein interactions. In particular, the HGC uses protein–protein interaction data from the String database (https://string-db.org/), considering only binding interactions that have been previously described. It has been shown in previous studies that the HGC is an effective tool for predicting novel disease-causing genes, according to their biological distance to known disease-causing genes^6–9^. Here we extended the HGC approach to increase the list of known BAT pathway genes into predicted BAT genes.

The HGC and its associated server^10^ could be used to detect new BAT candidate genes, by ranking all human genes on the basis of their biological proximity to already known BAT genes (i.e., genes relevant for BAT function), assuming the most highly ranked genes to be the most likely related to BAT. A list of novel potential BAT candidate genes, rigorously identified on the basis of biological relevance to BAT phenotype, would be useful and easy for most BAT investigators to use.

We generated a list of in silico-predicted novel human BAT candidate genes. We considered uncoupling protein 1 (UCP1) gene as the unique core gene. UCP1 is considered a hallmark of brown adipocyte, and is involved in the uncoupling of the electron-transport chain in the mitochondria inner membrane^1^. Therefore, its relevance and exclusivity on brown adipocytes is beyond doubt. Genes biologically close to UCP1 should therefore be strong candidates for being BAT-associated. Consequently, we used the HGC to extract the top 1% of human genes biologically closest to UCP1. We found that 60 genes already associated with BAT were included in that list, being the rest of them (107) the set of novel candidates.

## Material and methods

### Extraction of BAT connectome

We used, as an input, the connectome of UCP1, being the set of all human genes ranked according to their biological proximity to UCP1. These biological distances are predicted using protein–protein interaction patterns^5^. From this connectome, we extracted only the genes in the top 1% of all human genes by p-value and obtained a list of 168 genes (167 excluding UCP1 itself). We considered these genes as the connectome of BAT (herein called BAT connectome).

### Filtering of BAT candidate genes

We searched in PubMed and Web of Science for studies analysing the association of any of the 167 identified genes and BAT (known BAT genes hereafter). We considered as novel candidates those genes for which we found no evidence of relationship with BAT (candidate BAT genes hereafter). We are aware that some genes with a relationship described with BAT could not have been included in our review of BAT genes. However, an increase in the number of known BAT genes into the connectome would further support its predictive power and the relevance of the remaining candidates (see Supplementary Appendix S1 for further details about the search).

### Phylogeny of BAT genes

The biological-interrelatedness between known and candidate BAT genes was estimated with the functional genomic alignment phylogeny^5^. We first created a biological distance matrix between all BAT genes. Then, we applied a neighbour-joining algorithm by means of the *nj* function of the R package APE^11^. Finally, we plotted a fan-shaped tree based on HGC-predicted biological distances between BAT genes, using the plot function of APE package^11^. Note that this tree does not represent evolutionary relationships between genes (i.e., molecular evolutionary genetic distance), but their aggrupation based on the functional biological distance between them.

### Assessment of the predictive power

We assessed the predictive power of BAT connectome by testing whether the known BAT genes included in the connectome were significantly closer to each other than to other genes. First, we calculated the median distance between known BAT genes (3540 distances considered). Then, we sampled 1 million random sets of 3540 biological distances between known BAT genes and other genes. A p-value was estimated by determining the proportion of simulated random gene sets with a median distance to the known BAT genes equal or smaller to the distance between known BAT genes.

We further evaluated the predictive power of the connectome by testing whether known BAT genes were biologically closer to BAT candidates compared to other human genes. The median distance between candidate and known BAT genes inside the connectome (based on 6420 distances) was compared with the median distance between known BAT and random genes (1 million sets of 6420 random distances). Then, we estimated a p-value by determining the proportion of random sets with a median biological distance equal or smaller to the distance between candidate and known BAT genes. These and previous analyses were performed with R^12^.

### Gene expression analyses

We further validated our results using previously published microarray studies (gene expression related to BAT). We considered 4 microarray datasets that we were able to retrieve from ArrayExpress^13^ through the “ArrayExpress” R package^14^ and included raw data in order to be processed (see below). Two of these studies analyzed biopsies from human brown adipose tissue in the deep neck (around the carotid sheath) and the thyroid gland (close to the isthmus region), respectively^15,16^⁠. The other two studies generated brown adipocytes from human mesenchymal stem cells, induced pluripotent stem cells or fibroblasts^17,18^⁠. In all studies, we considered only those groups of arrays (i.e., samples) that showed BAT features like the expression of BAT markers (e.g., UCP1 and cell death-inducing DFFA-like effector a (CIDEA)), an increase of mitochondrial content, etc^15–18^. We only considered studies for which raw microarray data was available in order to perform the same processing procedure for all datasets. In particular, we applied the Robust Multichip Average processing methodology as implemented by the “oligo” R package^19^. After the processing, we analyzed the quality of the arrays using the “arrayQualityMetrics” R package^20^. We removed those samples identified as outliers and/or close to controls, i.e., non-BAT samples, in order to increase the probability of retaining only BAT-like samples. In any case, we compared the results with and without these samples and we found no qualitative differences (data not shown). Then, we matched expression data from the microarrays probes to specific genes using the “AnnotationDbi” R package^21^. We removed any probe associated with several genes, as it could not be ambiguously annotated. For the remaining probes, we calculated the median expression of all probes associated with each gene in order to get an average value of expression per gene. In this way, we obtained a value of expression for around 20,000 genes in each study. We ranked genes based on their expression in each study and selected those with an expression higher than the 95th percentile. Finally, we count the number of BAT connectome genes that were in the top of expression in any of the studies considered. We repeated this process with 1 million random sets of genes in order to test the significance of the results. A p-value was estimated by determining the proportion of random sets having a number of genes in the top of expression equal or greater than the BAT connectome.

## Results

### Final set of proposed novel BAT genes

**Figure 1:**
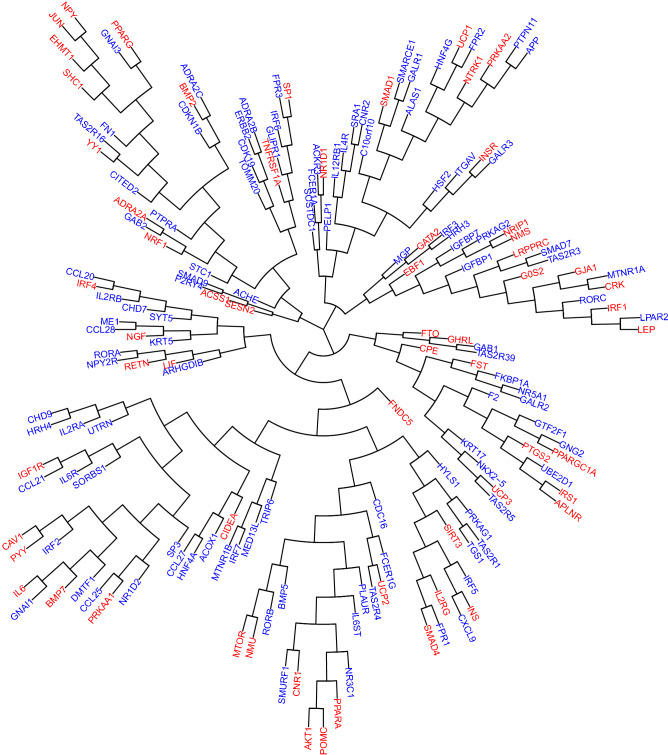
Functional genomic alignment phylogeny of brown adipose tissue (BAT) genes. A tree of biological distances generated by the functional genomic alignment method, showing hierarchical clustering of BAT genes. The genes related to BAT according to the literature search are shown in red, whilst predicted-novel candidate genes are shown in blue.

We found 60 genes that were already known to be associated with BAT inside the connectome, leaving a final list of 107 candidate genes (Tables 1, 2, Supplementary Appendix S1, S2). All these genes were included in a hierarchical clustering^5,11^. This analysis showed that the BAT candidate genes identified in this study were evenly distributed over the whole range of known BAT genes (Fig. 1).

**Table 1:** List of known-BAT genes. See Supplementary Appendix S1 for full gene names and details about the literature search performed to generate this list.

Known-BAT genes
ACSS1
ADRA2A
AKT1
APLNR
BMP2
BMP7
CAV1
CIDEA
CNR1
CPE
CRK
EBF1
EHMT1
FNDC5
FST
FTO
G0S2
GATA2
GHRL
GJA1
IGF1R
IL2RG
IL6
INS
INSR
IRF1
IRF4
IRS1
JUN
LEP
LIF
LRPPRC
MTOR
NGF
NMS
NMU
NPY
NR1D1
NRF1
NRIP1
NTRK1
POMC
PPARA
PPARG
PPARGC1A
PRKAA1
PRKAA2
PTGS2
PYY
RETN
SESN2
SHC1
SIRT3
SMAD1
SMAD4
SP1
TNFRSF1A
UCP1
UCP2
UCP3
YY1

**Table 2:** List of BAT candidates, along with information about their expression in two microarray studies analyzing BAT biopsies^15,16^. 1 = Above the 95th percentile of gene expression in any of the two studies; 0 = Below the 95th percentile of gene expression in both studies. See Supplementary Appendix S1 for full gene names.

BAT candidates	Highly expressed in BAT biopsies
ACHE	0
ACKR3	0
ACOX1	0
ADRA2B	0
ADRA2C	0
ALAS1	0
APP	1
ARHGDIB	0
BMP5	0
C10orf10	0
CCL20	0
CCL21	0
CCL25	0
CCL27	0
CCL28	0
CDC16	1
CDK19	0
CDKN1B	1
CHD7	0
CHD9	0
CITED2	1
CNR2	0
CXCL9	0
DMTF1	1
ERBB2	0
F2	0
FCER1A	0
FCER1G	0
FKBP1A	1
FN1	1
FPR1	0
FPR2	0
FPR3	0
GAB1	0
GAB2	0
GALR1	0
GALR2	0
GALR3	0
GLIPR1	1
GNAI1	0
GNAI3	0
GNG2	0
GTF2F1	0
HNF4A	0
HNF4G	0
HRH3	0
HRH4	0
HSF2	0
HYLS1	0
IGFBP1	0
IGFBP7	1
IL12RB1	0
IL2RA	0
IL2RB	0
IL4R	0
IL6R	0
IL6ST	1
IRF2	0
IRF3	0
IRF5	0
IRF6	0
IRF7	0
ITGAV	1
KRT17	0
KRT5	0
LPAR2	0
ME1	0
MED13L	1
MGP	1
MTNR1A	0
MTNR1B	0
NKX2-5	0
NPY2R	0
NR1D2	0
NR3C1	1
NR5A1	0
P2RY4	0
PELP1	0
PLAUR	0
PRKAG1	0
PRKAG2	0
PTPN11	0
PTPRA	0
RORA	0
RORB	0
RORC	0
SMAD7	0
SMAD9	0
SMARCE1	0
SMURF1	0
SORBS1	0
SOSTDC1	0
SP3	0
SRA1	0
STC1	0
SYT5	0
TAS2R1	0
TAS2R16	0
TAS2R3	0
TAS2R39	0
TAS2R4	0
TAS2R5	0
TGS1	0
TOMM20	1
TRIP6	0
UBE2D1	0
UTRN	0

### Assessment of predictive power

We found that the median biological distance between known BAT genes inside the connectome was 4.72. None of the simulated sets of random genes had a median distance to known BAT genes equal or smaller than 4.72 (p < 2.2 × 10^–16^; 95 CI 0–4.79 × 10^–6^). Known BAT genes were also biologically closer to BAT candidates compared to random set of genes that were outside of the connectome (p < 2.2 × 10^–16^; 95 CI 0–4.79 × 10^–6^). These results are consistent with the hypothesis of tight functional interrelatedness between BAT genes. It was expected that genes belonging to the same pathway would display small biological distances between each other. Confirming this hypothesis makes it possible to infer novel genes related to BAT based on HGC-predicted biological distances to known BAT genes.

### Gene expression analyses

The validation analysis using gene expression data related to BAT showed that 37 BAT connectome genes were included in the top of expression (95th percentile) of any of the 4 studies considered. This number was higher than expected by chance (p = 2.2 × 10^–04^; 95 CI 1.41 × 10^–04^–3.39 × 10^–04^), suggesting that the genes included in the BAT connectome tend to be more expressed in BAT than expected under the null expectation. When focusing on BAT-candidates, we found that 18 candidates were in the top of expression of the studies considered, being this number only marginally significant (p = 1.01 × 10^–01^; 95 CI 9.95 × 10^–02^–1.03 × 10^–01^). This lack of significance is possibly caused by a lack of power. This is supported by the fact that only 19 known-BAT genes were present in the top of expression (p = 8 × 10^–^^05^; 95 CI 3.72 × 10^–05^–1.64 × 10^–04^). In other words, there were still 42 known-BAT genes not included in the BAT top of expression. Note that all these genes are known to be implicated in BAT functioning. Therefore, although these analyses detected a signal of association with BAT, they did not have enough power to detect the full connection between these genes and BAT expression, thus being conservative. We hypothesized that the experimental procedures used to differentiate cells to brown adipocytes could introduce noise, so we repeated the analyses but considering only the two studies analyzing biopsies of brown adipose tissue. Considering these studies, we found even stronger support for the enrichment of BAT-expressed genes within the BAT connectome (p = 3 × 10^–05^; 95 CI 7.75 × 10^–06^–9.56 × 10^–05^). This is congruent with an increase of power after focusing specifically on actual BAT biopsies. Interestingly, the signal also becomes stronger in the subset of BAT candidates, existing a significant enrichment of genes expressed in BAT among these candidates (p = 4.39 × 10^–02^; 95 CI 4.26 × 10^–02^–4.51 × 10^–02^). In other words, the BAT candidates also tend to be more expressed in BAT than expected by chance when focusing on the gene expression of BAT biopsies (see Table 2 for the list of BAT candidates present in the top expression of BAT biopsies studies). These results support the functional connection of the BAT connectome with brown adipose tissue and the relevance of the BAT candidates as novel genes potentially implicated in the functioning of this tissue.

## Discussion

We described here a list of 107 novel human BAT genes candidates, initially predicted in silico with the HGC biological distance concept. We extracted 1% of genes biologically closest to UCP1. From this connectome, we selected those genes for which there was no previous evidence of relationship with BAT. We generated a final in silico-predicted set of 107 human genes, which may be considered reliable BAT candidate genes. In other words, we predicted that there will be a high proportion of BAT genes among these 107 genes. We demonstrated the predictive power of the BAT connectome by showing that known BAT genes were biologically closer to each other than expected by chance. In addition, known BAT genes were closer to BAT candidates compared to random sets of genes. Finally, we found that the BAT connectome as a whole and the BAT-candidates tend to be more expressed in BAT biopsies than expected under the null expectation, supporting the functional connection between these genes and BAT. The resulting new list of predicted human BAT genes should be useful for the discovery of novel BAT genes and related metabolic pathways.

Evidence about their implication in adipose tissue thermogenesis has recently emerged for some of these BAT candidate genes. This is the case of the Grb2-associated binding protein 2 (GAB2), a protein implicated in amplification and integration of signalling pathways in the cytoplasm. The HGC predicted that GAB2 is biologically close to UCP1, but no evidence supported a direct link between GAB2 and UCP1 or BAT at the moment our analyses were done. Wang et al.^22^ have recently reported evidence supporting this link. Compared to the wildtype, BAT from Gab2-KO mice has a higher expression of *Ucp1* and other thermogenic genes (e.g., *Cidea* and peroxisome proliferator-activated receptor-gamma coactivator 1-alpha (*Pgc-1a*)). Interestingly, *Ucp1* was not more expressed in the inguinal white adipose tissue (WAT) from Gab2-KO mice, suggesting that Gab2 is BAT-specific. They also showed that Gab2 has an impact in brown-adipocyte differentiation. The deletion of Gab2 increased the transcription of *Ucp1* in brown preadipocytes, leading to a higher Ucp1 content at the end of differentiation along with higher expression of other thermogenic genes. Therefore, recent evidence suggests a role of GAB2 in the development and functioning of BAT, as predicted by the BAT connectome. The histamine H4 receptor (HRH4), one of the proteins that mediates the effect of histamine, is another BAT candidate. The HGC also predicted this protein to be biologically close to UCP1, but again, no evidence supported a direct connection until recently. Zhao et al.^23^ has reported an increase of HrH4 expression in the subcutaneous WAT of mice exposed to cold conditions. They also reported an impairment of cold-induced browning in WAT of HrH4-KO mice. KO mice showed an impaired up-regulation of thermogenic genes, including *Ucp1* and *Pgc-1α*, along with a worse regulation of temperature and lower heat production in response to cold. Interestingly, the treatment with 4-methylhistamine, an specific agonist of HrH4, increased the browning of subcutaneous WAT in mice. This suggests a role of HRH4 as a novel regulator of browning and cold-induced thermogenesis in white adipose tissue. Therefore, this protein might also play a role in UCP1-related thermogenesis in BAT, as predicted by the HGC. In summary, recent evidence is starting to support the predictions of the BAT connectome.

There are BAT candidates with no direct link to BAT but for which current evidence suggests that the association is plausible. Insulin-like growth factor-binding protein 7 gene (*IGFBP7*) would be an example of this. According to the HGC, IGFBP7 interacts with C-Jun (*JUN*) that in turn interacts with UCP1, thus being IGFBP7 biologically close to UCP1. However, there is currently no direct link between IGFBP7 and UCP1. In this regard, Hu et al.^24^ analyzed the impact of IGFBP7 on cattle subcutaneous preadipocytes. They found that knockout preadipocytes for *IGFBP7* had lower lipid accumulation and triglycerides formation during maturation compared to controls. In addition, these knockout adipocytes showed lower expression of adipogenic-related genes. Conversely, cattle preadipocytes overexpressing *IGFBP7* showed the opposite pattern, i.e., higher lipid accumulation, triglyceride formation and expression of adipogenic-related genes. One of these adipogenic genes was the Peroxisome proliferator-activated receptor gamma gene (*PPARG*), which is also included in the BAT connectome as a known-BAT gene. Therefore, *IGFBP7* may be also implicated in the differentiation of brown adipocytes. The integrin alpha-V gene (*ITGAV*) would be another example of a potential BAT gene that has not been directly associated with BAT yet. ITGAV interacts with the Insulin receptor substrate 1 (IRS1) according to the HGC, which in turn interacts with UCP1, but no direct link to brown adipose tissue has been reported. Morandi et al.^25^ studied the role of ITGAV in the differentiation of adipose derived stem cells obtained from human subcutaneous adipose tissue. They found that the silencing of *ITGAV* enhanced adipogenic differentiation. In contrast, *ITGAV* overexpression decreased the mRNA levels of adipocyte marker genes, namely *Adiponectin* (*ADIPOQ*) and *PPARG*. They also tested the impact of an *ITGAV* inhibitor (Cilengitide). As expected, it influenced adipocyte differentiation in a similar way than the silencing of *ITGAV*, i.e., enhancement of adipogenesis and strongly induction of PPARG, which is a known-BAT gene in the BAT connectome. Therefore, *ITGAV* seems to be implicated in white adipocyte differentiation and may be also relevant in the differentiation of brown adipocytes. These results, along with the fact that both *IGFBP7* and *ITGAV* are above the 95th percentile of expression in human BAT biopsies, support the relevance of these genes as strong candidates for BAT functioning and development.

It is important to note that, as in any high-throughput genome-wide analysis, the choice of input data, algorithm, and even small fine-tuning is likely to strongly affect the outcome. However, we attempted to provide a reliable prediction where false positives (i.e., false BAT genes that were included in the final provided list of candidate genes) were minimized—an aim that is expected to be achieved by selecting only one core gene strongly implicated with the functioning of BAT, i.e., UCP1. This approach has as counterpart a higher probability of false negatives (i.e., true BAT genes that were not included in the final list), but we preferred to prioritise the exclusion of false positives given the type of study for which this connectome is mainly aimed: candidate gene association studies. These type of studies use a limited list of genes whose relationship with interest phenotype has to be suggested by previous knowledge^4^. Therefore, a list of novel-candidate genes that are predicted to be biologically close to known BAT genes increases the potential list of genes to be used in candidate gene association studies, and hence could be useful to improve our knowledge of the genetic architecture of human BAT.

## Supplementary Information

Supplementary Information.

## Data Availability

The programs and online server used in this study (in particular for ranking candidate genes by biological distance from UCP1 and the generation of functional genomic alignment trees) are freely available to non-commercial users with step-by-step instructions at http://lab.rockefeller.edu/casanova/HGC and http://hgc.rockefeller.edu. The rest of scripts for the technical procedures in this study are available upon request.

## Abbreviations

BAT: Brown adipose tissue
HGC: Human genome connectome
UCP1: Uncoupling protein 1
GAB2: Grb2-associated binding protein 2
CIDEA: Cell death-inducing DFFA-like effector a
PGC1A: Peroxisome proliferator-activated receptor gamma coactivator 1-alpha
HRH4: Histamine H4 receptor
IGFBP7: Insulin-like growth factor-binding protein 7
PPARG: Peroxisome proliferator-activated receptor gamma
ITGAV: Integrin alpha-V
IRS1: Insulin receptor substrate 1
ADIPOQ: Adiponectin
