# Supplementary material for: Novel brown adipose tissue candidate genes predicted by the human gene connectome
Source: Sci Rep. 2022 May 9;12:7614. doi: 10.1038/s41598-022-11317-2 (PMC9085833; doi:10.1038/s41598-022-11317-2)
Supplement: Supplementary file 1 — Supplementary Information. [file 41598_2022_11317_MOESM1_ESM.docx]

**Supplementary Appendix**

**Title:** Novel brown adipose tissue candidate genes predicted by the human gene connectome

**List of authors:** Diego F. Salazar-Tortosa^1,2*^, David Enard^2^, Yuval Itan^3,4^, Jonatan R. Ruiz^1,5,6*^

*corresponding authors

**Institutions:**

^1^PROFITH ‘PROmoting FITness and Health through physical activity’ research group, Sport and Health University Research Institute (iMUDS), University of Granada, Granada, Spain.

^2^Department of Ecology and Evolutionary Biology, University of Arizona, Tucson, Arizona, USA.

^3^The Charles Bronfman Institute of Personalized Medicine, Icahn School of Medicine at Mount Sinai, New York, NY, USA.

^4^Department of Genetics and Genomic Sciences, Icahn School of Medicine at Mount Sinai, New York, NY, USA.

^5^Department of Physical Education and Sport, Faculty of Sport Sciences, University of Granada, Granada, Spain.

^6^Instituto de Investigación Biosanitaria, ibs.Granada, Granada, Spain.

**Supplementary Appendix S1:** List of genes included in the brown adipose tissue (BAT) connectome, along with information about their relationship with BAT. Abbreviations: 0 = Predicted-novel BAT candidate genes; 1 = Known relationship. The information about BAT relationship was obtained by doing the following search for each gene: (((("Adipose Tissue, Brown"[Mesh] OR "Brown Fat" OR "Brown adipose tissue"))) OR (("Adipose tissue, beige"[Mesh] OR "beige adipose tissue" OR "Brite fat" OR "beige fat"))) AND (GENE NAME). Note that some genes with a relationship described with BAT could not have been included in this review. However, an increase in the number of known BAT genes in the connectome would further support its predictive power and the relevance of the remaining candidates.

| **Genes** | **Description** | **BAT** | **References** |
| --- | --- | --- | --- |
|  |  | **relationship** |  |
| ACHE | Acetylcholinesterase | 0 |  |
| ACKR3 | Atypical chemokine receptor 3 | 0 |  |
| ACOX1 | Acyl-CoA oxidase 1 | 0 |  |
| ACSS1 | Acyl-CoA synthetase short chain family member 1 | 1 | ^1^ |
| ADRA2A | Adrenoceptor alpha 2A | 1 | ^2^ |
| ADRA2B | Adrenoceptor alpha 2B | 0 |  |
| ADRA2C | Adrenoceptor alpha 2C | 0 |  |
| AKT1 | AKT serine/threonine kinase 1 | 1 | ^3^ |
| ALAS1 | 5'-aminolevulinate synthase 1 | 0 |  |
| APLNR | Apelin receptor | 1 | ^4^ |
| APP | Amyloid beta precursor protein | 0 |  |
| ARHGDIB | Rho GDP dissociation inhibitor beta | 0 |  |
| BMP2 | Bone morphogenetic protein 2 | 1 | ^5^ |
| BMP5 | Bone morphogenetic protein 5 | 0 |  |
| BMP7 | Bone morphogenetic protein 7 | 1 | ^6^ |
| C10orf10 | Chromosome 10 open reading frame 10 | 0 |  |
| CAV1 | Caveolin 1 | 1 | ^7^ |
| CCL20 | C-C motif chemokine ligand 20 | 0 |  |
| CCL21 | C-C motif chemokine ligand 21 | 0 |  |
| CCL25 | C-C motif chemokine ligand 25 | 0 |  |
| CCL27 | C-C motif chemokine ligand 27 | 0 |  |
| CCL28 | C-C motif chemokine ligand 28 | 0 |  |
| CDC16 | Cell division cycle 16 | 0 |  |
| CDK19 | Cyclin dependent kinase 19 | 0 |  |
| CDKN1B | Cyclin dependent kinase inhibitor 1B | 0 |  |
| CHD7 | Chromodomain helicase DNA binding protein 7 | 0 |  |
| CHD9 | Chromodomain helicase DNA binding protein 9 | 0 |  |
| CIDEA | Cell death-inducing DFFA-like effector A | 1 | ^8^ |
| CITED2 | Cbp/p300 interacting transactivator with | 0 |  |
|  | Glu/Asp rich carboxy-terminal domain 2 |  |  |
| CNR1 | Cannabinoid receptor 1 | 1 | ^9^ |
| CNR2 | Cannabinoid receptor 2 | 0 |  |
| CPE | Carboxypeptidase E | 1 | ^10^ |
| CRK | CRK proto-oncogene, adaptor protein | 1 | ^11^ |
| CXCL9 | C-X-C motif chemokine ligand 9 | 0 |  |
| DMTF1 | Cyclin D binding myb like transcription factor 1 | 0 |  |
| EBF1 | Early B-cell factor 1 | 1 | ^12^ |
| EHMT1 | Euchromatic histone lysine methyltransferase 1 | 1 | ^13^ |
| ERBB2 | Erb-b2 receptor tyrosine kinase 2 | 0 |  |
| F2 | Coagulation factor II, thrombin | 0 |  |
| FCER1A | Fc fragment of IgE receptor Ia | 0 |  |
| FCER1G | Fc fragment of IgE receptor Ig | 0 |  |
| FKBP1A | FK506 binding protein 1A | 0 |  |
| FN1 | Fibronectin 1 | 0 |  |
| FNDC5 | Fibronectin type III domain containing 5 | 1 | ^14^ |
| FPR1 | Formyl peptide receptor 1 | 0 |  |
| FPR2 | Formyl peptide receptor 2 | 0 |  |
| FPR3 | Formyl peptide receptor 3 | 0 |  |
| FST | Follistatin | 1 | ^15^ |
| FTO | FTO, alpha-ketoglutarate dependent dioxygenase | 1 | ^16^ |
| G0S2 | G0/G1 switch 2 | 1 | ^17^ |
| GAB1 | GRB2 associated binding protein 1 | 0 |  |
| GAB2 | GRB2 associated binding protein 2 | 0 |  |
| GALR1 | Galanin receptor 1 | 0 |  |
| GALR2 | Galanin receptor 2 | 0 |  |
| GALR3 | Galanin receptor 3 | 0 |  |
| GATA2 | GATA binding protein 2 | 1 | ^18^ |
| GHRL | Ghrelin and obestatin prepropeptide | 1 | ^19^ |
| GJA1 | Gap junction protein alpha 1 | 1 | ^20^ |
| GLIPR1 | GLI pathogenesis related 1 | 0 |  |
| GNAI1 | G protein subunit alpha i1 | 0 |  |
| GNAI3 | G protein subunit alpha i3 | 0 |  |
| GNG2 | G protein subunit gamma 2 | 0 |  |
| GTF2F1 | General transcription factor IIF subunit 1 | 0 |  |
| HNF4A | Hepatocyte nuclear factor 4 alpha | 0 |  |
| HNF4G | Hepatocyte nuclear factor 4 gamma | 0 |  |
| HRH3 | Histamine receptor H3 | 0 |  |
| HRH4 | Histamine receptor H4 | 0 |  |
| HSF2 | Heat shock transcription factor 2 | 0 |  |
| HYLS1 | HYLS1, centriolar and ciliogenesis associated | 0 |  |
| IGF1R | Insulin like growth factor 1 receptor | 1 | ^21^ |
| IGFBP1 | Insulin like growth factor binding protein 1 | 0 |  |
| IGFBP7 | Insulin like growth factor binding protein 7 | 0 |  |
| IL12RB1 | Interleukin 12 receptor subunit beta 1 | 0 |  |
| IL2RA | Interleukin 2 receptor subunit alpha | 0 |  |
| IL2RB | Interleukin 2 receptor subunit beta | 0 |  |
| IL2RG | Interleukin 2 receptor subunit gamma | 1 | ^22^ |
| IL4R | Interleukin 4 receptor | 0 |  |
| IL6 | Interleukin 6 | 1 | ^23^ |
| IL6R | Interleukin 6 receptor | 0 |  |
| IL6ST | Interleukin 6 signal transducer | 0 |  |
| INS | Insulin | 1 | ^24^ |
| INSR | Insulin receptor | 1 | ^25^ |
| IRF1 | Interferon regulatory factor 1 | 1 | ^26^ |
| IRF2 | Interferon regulatory factor 2 | 0 |  |
| IRF3 | Interferon regulatory factor 3 | 0 |  |
| IRF4 | Interferon regulatory factor 4 | 1 | ^27^ |
| IRF5 | Interferon regulatory factor 5 | 0 |  |
| IRF6 | Interferon regulatory factor 6 | 0 |  |
| IRF7 | Interferon regulatory factor 7 | 0 |  |
| IRS1 | Insulin receptor substrate 1 | 1 | ^28^ |
| ITGAV | Integrin subunit alpha V | 0 |  |
| JUN | Jun proto-oncogene, AP-1 transcription factor subunit | 1 | ^29^ |
| KRT17 | Keratin 17 | 0 |  |
| KRT5 | Keratin 5 | 0 |  |
| LEP | Leptin | 1 | ^30^ |
| LIF | LIF, interleukin 6 family cytokine | 1 | ^31^ |
| LPAR2 | Lysophosphatidic acid receptor 2 | 0 |  |
| LRPPRC | Leucine rich pentatricopeptide repeat containing | 1 | ^32^ |
| ME1 | Malic enzyme 1 | 0 |  |
| MED13L | Mediator complex subunit 13 like | 0 |  |
| MGP | Matrix Gla protein | 0 |  |
| MTNR1A | Melatonin receptor 1A | 0 |  |
| MTNR1B | Melatonin receptor 1B | 0 |  |
| MTOR | Mechanistic target of rapamycin kinase | 1 | ^33^ |
| NGF | Nerve growth factor | 1 | ^34^ |
| NKX2-5 | NK2 homeobox 5 | 0 |  |
| NMS | Neuromedin S | 1 | ^35^ |
| NMU | Neuromedin U | 1 | ^35^ |
| NPY | Neuropeptide Y | 1 | ^36^ |
| NPY2R | Neuropeptide Y receptor Y2 | 0 |  |
| NR1D1 | Nuclear receptor subfamily 1 group D member 1 | 1 | ^37^ |
| NR1D2 | Nuclear receptor subfamily 1 group D member 2 | 0 |  |
| NR3C1 | Nuclear receptor subfamily 3 group C member 1 | 0 |  |
| NR5A1 | Nuclear receptor subfamily 5 group A member 1 | 0 |  |
| NRF1 | Nuclear respiratory factor 1 | 1 | ^38^ |
| NRIP1 | Nuclear receptor interacting protein | 1 | ^39^ |
| NTRK1 | Neurotrophic receptor tyrosine kinase 1 | 1 | ^34^ |
| P2RY4 | Pyrimidinergic receptor P2Y4 | 0 |  |
| PELP1 | Proline, glutamate and leucine rich protein 1 | 0 |  |
| PLAUR | Plasminogen activator, urokinase receptor | 0 |  |
| POMC | Proopiomelanocortin | 1 | ^40^ |
| PPARA | Peroxisome proliferator activated receptor alpha | 1 | ^41^ |
| PPARG | Peroxisome proliferator activated receptor gamma | 1 | ^42^ |
| PPARGC1A | PPARG coactivator 1 alpha | 1 | ^43^ |
| PRKAA1 | Protein kinase AMP-activated catalytic subunit alpha 1 | 1 | ^9^ |
| PRKAA2 | Protein kinase AMP-activated catalytic subunit alpha 2 | 1 | ^44^ |
| PRKAG1 | Protein kinase AMP-activated non-catalytic subunit gamma 1 | 0 |  |
| PRKAG2 | Protein kinase AMP-activated non-catalytic subunit gamma 2 | 0 |  |
| PTGS2 | Prostaglandin-endoperoxide synthase 2 | 1 | ^45^ |
| PTPN11 | Protein tyrosine phosphatase, non-receptor type 11 | 0 |  |
| PTPRA | Protein tyrosine phosphatase, receptor type A | 0 |  |
| PYY | Peptide YY | 1 | ^46^ |
| RETN | Resistin | 1 | ^47^ |
| RORA | RAR related orphan receptor A | 0 |  |
| RORB | RAR related orphan receptor B | 0 |  |
| RORC | RAR related orphan receptor C | 0 |  |
| SESN2 | Sestrin 2 | 1 | ^48^ |
| SHC1 | SHC adaptor protein 1 | 1 | ^49^ |
| SIRT3 | Sirtuin 3 | 1 | ^50^ |
| SMAD1 | SMAD family member 1 | 1 | ^51^ |
| SMAD4 | SMAD family member 4 | 1 | ^51^ |
| SMAD7 | SMAD family member 7 | 0 |  |
| SMAD9 | SMAD family member 9 | 0 |  |
| SMARCE1 | SWI/SNF related, matrix associated, actin dependent | 0 |  |
|  | regulator of chromatin, subfamily e, member 1 |  |  |
| SMURF1 | SMAD specific E3 ubiquitin protein ligase 1 | 0 |  |
| SORBS1 | Sorbin and SH3 domain containing 1 | 0 |  |
| SOSTDC1 | Sclerostin domain containing 1 | 0 |  |
| SP1 | Sp1 transcription factor | 1 | ^52^ |
| SP3 | Sp3 transcription factor | 0 |  |
| SRA1 | Steroid receptor RNA activator 1 | 0 |  |
| STC1 | Stanniocalcin 1 | 0 |  |
| SYT5 | Synaptotagmin 5 | 0 |  |
| TAS2R1 | Taste 2 receptor member 1 | 0 |  |
| TAS2R16 | Taste 2 receptor member 16 | 0 |  |
| TAS2R3 | Taste 2 receptor member 3 | 0 |  |
| TAS2R39 | Taste 2 receptor member 39 | 0 |  |
| TAS2R4 | Taste 2 receptor member 4 | 0 |  |
| TAS2R5 | Taste 2 receptor member 5 | 0 |  |
| TGS1 | Trimethylguanosine synthase 1 | 0 |  |
| TNFRSF1A | TNF receptor superfamily member 1A | 1 | ^53^ |
| TOMM20 | Translocase of outer mitochondrial membrane 20 | 0 |  |
| TRIP6 | Thyroid hormone receptor interactor 6 | 0 |  |
| UBE2D1 | Ubiquitin conjugating enzyme E2 D1 | 0 |  |
| UCP1 | Uncoupling protein 1 | 1 | ^54^ |
| UCP2 | Uncoupling protein 2 | 1 | ^55^ |
| UCP3 | Uncoupling protein 3 | 1 | ^56^ |
| UTRN | Utrophin | 0 |  |
| YY1 | YY1 transcription factor | 1 | ^57^ |

**Supplementary Appendix S2:** Known brown adipose tissue (BAT) genes included in the BAT connectome along with distance, rank, p-value, and route respect to uncoupling protein 1, core gene of the connectome.

| **Genes** | **Biological** | **Rank in BAT** | **p-Value** | **Route to core** |
| --- | --- | --- | --- | --- |
|  | **distance to core** | **connectome** | **(percentile)** |  |
| UCP2 | 1.11 | 1 | 0.00006 | UCP1↔UCP2 |
| UCP3 | 1.11 | 2 | 0.00012 | UCP1↔UCP3 |
| BMP7 | 1.25 | 3 | 0.00018 | UCP1↔BMP7 |
| FNDC5 | 1.25 | 4 | 0.00024 | UCP1↔FNDC5 |
| CIDEA | 1.25 | 5 | 0.00030 | UCP1↔CIDEA |
| INS | 1.25 | 6 | 0.00036 | UCP1↔INS |
| IRS1 | 1.25 | 7 | 0.00042 | UCP1↔IRS1 |
| IL6 | 1.25 | 8 | 0.00048 | UCP1↔IL6 |
| RETN | 1.25 | 9 | 0.00054 | UCP1↔RETN |
| JUN | 1.25 | 10 | 0.00060 | UCP1↔JUN |
| SIRT3 | 1.25 | 11 | 0.00066 | UCP1↔SIRT3 |
| LRPPRC | 1.25 | 12 | 0.00072 | UCP1↔LRPPRC |
| IGF1R | 1.25 | 13 | 0.00078 | UCP1↔IGF1R |
| NRIP1 | 1.25 | 14 | 0.00084 | UCP1↔NRIP1 |
| FTO | 1.25 | 15 | 0.00090 | UCP1↔FTO |
| SESN2 | 1.25 | 16 | 0.00096 | UCP1↔SESN2 |
| GHRL | 1.25 | 17 | 0.00102 | UCP1↔GHRL |
| PPARGC1A | 1.25 | 18 | 0.00108 | UCP1↔PPARGC1A |
| PPARG | 1.25 | 19 | 0.00114 | UCP1↔PPARG |
| PPARA | 1.25 | 20 | 0.00119 | UCP1↔PPARA |
| LEP | 1.25 | 21 | 0.00125 | UCP1↔LEP |
| NPY | 1.25 | 22 | 0.00131 | UCP1↔NPY |
| SP1 | 4.72 | 24 | 0.00143 | UCP1↔JUN↔SP1 |
| SMAD4 | 4.72 | 30 | 0.00179 | UCP1↔UCP2↔SMAD4 |
| SMAD1 | 4.72 | 32 | 0.00191 | UCP1↔BMP7↔SMAD1 |
| GJA1 | 4.72 | 36 | 0.00215 | UCP1↔JUN↔GJA1 |
| APLNR | 4.72 | 37 | 0.00221 | UCP1↔NPY↔APLNR |
| NR1D1 | 4.72 | 38 | 0.00227 | UCP1↔PPARG↔NR1D1 |
| AKT1 | 4.72 | 40 | 0.00239 | UCP1↔NRIP1↔AKT1 |
| NMU | 4.72 | 49 | 0.00293 | UCP1↔NPY↔NMU |
| NMS | 4.72 | 50 | 0.00299 | UCP1↔NPY↔NMS |
| PTGS2 | 4.72 | 52 | 0.00311 | UCP1↔JUN↔PTGS2 |
| NTRK1 | 4.72 | 53 | 0.00317 | UCP1↔IRS1↔NTRK1 |
| BMP2 | 4.72 | 55 | 0.00329 | UCP1↔BMP7↔BMP2 |
| IL2RG | 4.72 | 75 | 0.00448 | UCP1↔IRS1↔IL2RG |
| LIF | 4.72 | 82 | 0.00490 | UCP1↔JUN↔LIF |
| ADRA2A | 4.72 | 83 | 0.00496 | UCP1↔NPY↔ADRA2A |
| PRKAA2 | 4.72 | 93 | 0.00556 | UCP1↔UCP3↔PRKAA2 |
| PRKAA1 | 4.72 | 94 | 0.00562 | UCP1↔UCP2↔PRKAA1 |
| EBF1 | 4.72 | 97 | 0.00580 | UCP1↔PPARG↔EBF1 |
| SHC1 | 4.72 | 99 | 0.00592 | UCP1↔LRPPRC↔SHC1 |
| FST | 4.72 | 106 | 0.00633 | UCP1↔BMP7↔FST |
| ACSS1 | 4.72 | 109 | 0.00651 | UCP1↔SIRT3↔ACSS1 |
| INSR | 4.72 | 110 | 0.00657 | UCP1↔IRS1↔INSR |
| CNR1 | 4.72 | 112 | 0.00669 | UCP1↔NPY↔CNR1 |
| GATA2 | 4.72 | 122 | 0.00729 | UCP1↔JUN↔GATA2 |
| PYY | 4.72 | 123 | 0.00735 | UCP1↔NPY↔PYY |
| NGF | 4.72 | 125 | 0.00747 | UCP1↔IRS1↔NGF |
| G0S2 | 4.72 | 126 | 0.00753 | UCP1↔PPARA↔G0S2 |
| CAV1 | 4.72 | 128 | 0.00765 | UCP1↔IRS1↔CAV1 |
| YY1 | 4.72 | 132 | 0.00789 | UCP1↔PPARGC1A↔YY1 |
| POMC | 4.72 | 140 | 0.00836 | UCP1↔NPY↔POMC |
| CPE | 4.72 | 143 | 0.00854 | UCP1↔INS↔CPE |
| IRF1 | 4.72 | 153 | 0.00914 | UCP1↔JUN↔IRF1 |
| IRF4 | 4.72 | 157 | 0.00938 | UCP1↔JUN↔IRF4 |
| MTOR | 4.72 | 158 | 0.00944 | UCP1↔PPARGC1A↔MTOR |
| NRF1 | 4.72 | 159 | 0.00950 | UCP1↔UCP2↔NRF1 |
| CRK | 4.72 | 165 | 0.00986 | UCP1↔IRS1↔CRK |
| TNFRSF1A | 4.72 | 166 | 0.00992 | UCP1↔PPARG↔TNFRSF1A |
| EHMT1 | 4.72 | 167 | 0.00998 | UCP1↔IL6↔EHMT1 |

**References**

1. Nakamura, Y., Sato, T., Shiimura, Y., Miura, Y. & Kojima, M. FABP3 and brown adipocyte-characteristic mitochondrial fatty acid oxidation enzymes are induced in beige cells in a different pathway from UCP1. *Biochem. Biophys. Res. Commun.* **441**, 42–46 (2013).

2. Wang, Y., Fälting, J. M., Mattsson, C. L., Holmström, T. E. & Nedergaard, J. In brown adipocytes, adrenergically induced β1-/β3-(Gs)-, α2-(Gi)- and α1-(Gq)-signalling to Erk1/2 activation is not mediated via EGF receptor transactivation. *Exp. Cell Res.* **319**, 2718–2727 (2013).

3. Wu, R. *et al.* DJ-1 maintains energy and glucose homeostasis by regulating the function of brown adipose tissue. *Cell Discov.* **3**, 16054 (2017).

4. Than, A. *et al.* Apelin enhances brown adipogenesis and browning of white adipocytes. *J. Biol. Chem.* **290**, 14679–14691 (2015).

5. Olmsted-Davis, E. *et al.* Hypoxic adipocytes pattern early heterotopic bone formation. *Am. J. Pathol.* **170**, 620–32 (2007).

6. Seale, P. Transcriptional regulatory circuits controlling brown fat development and activation. *Diabetes* **64**, 2369–2375 (2015).

7. Cohen, A. W., Schubert, W., Brasaemle, D. L., Scherer, P. E. & Lisanti, M. P. Caveolin-1 expression is essential for proper nonshivering thermogenesis in brown adipose tissue. *Diabetes* **54**, 679–686 (2005).

8. Nishimoto, Y. & Tamori, Y. CIDE Family-Mediated Unique Lipid Droplet Morphology in White Adipose Tissue and Brown Adipose Tissue Determines the Adipocyte Energy Metabolism. *J Atheroscler Thromb* **24**, 0–0 (2017).

9. Monge-Roffarello, B. *et al.* The PVH as a site of CB1-mediated stimulation of thermogenesis by MC4R agonism in male rats. *Endocrinology* **155**, 3448–3458 (2014).

10. Collins, S., Daniel, K. W. & Rohlfs, E. M. Depressed expression of adipocyte beta-adrenergic receptors is a common feature of congenital and diet-induced obesity in rodents. *Int. J. Obes.* **23**, 669–77 (1999).

11. Miura, A. *et al.* Insulin substrates 1 and 2 are corequired for activation of atypical protein kinase C and Cbl-dependent phosphatidylinositol 3-kinase during insulin action in immortalized brown adipocytes. *Biochemistry* **43**, 15503–15509 (2004).

12. Kang, S. *et al.* Regulation of Early Adipose Commitment by Zfp521. *PLoS Biol.* **10**, (2012).

13. Nagano, G. *et al.* Activation of classical brown adipocytes in the adult human perirenal depot is highly correlated with PRDM16-EHMT1 complex expression. *PLoS One* **10**, 1–13 (2015).

14. Ge, X. *et al.* Myostatin signals through miR-34a to regulate Fndc5 expression and browning of white adipocytes. *Int. J. Obes.* **41**, 137–148 (2017).

15. Singh, R. *et al.* Follistatin targets distinct pathways to promote brown adipocyte characteristics in brown and white adipose tissues. *Endocrinology* **158**, 1217–1230 (2017).

16. Tews, D. *et al.* FTO deficiency induces UCP-1 expression and mitochondrial uncoupling in adipocytes. *Endocrinology* **154**, 3141–3151 (2013).

17. Fujimoto, Y. *et al.* TFE3 controls lipid metabolism in adipose tissue of male mice by suppressing lipolysis and thermogenesis. *Endocrinology* **154**, 3577–3588 (2013).

18. Tsai, J. *et al.* The transcription factor GATA2 regulates differentiation of brown adipocytes. *EMBO Rep.* **6**, 879–884 (2005).

19. Ma, X. *et al.* Ablations of ghrelin and ghrelin receptor exhibit differential metabolic phenotypes and thermogenic capacity during aging. *PLoS One* **6**, (2011).

20. Yan, J. *et al.* Detection of differential gene expression in brown adipose tissue of hibernating arctic ground squirrels with mouse microarrays. 346–353 (2006) doi:10.1152/physiolgenomics.00260.2005.

21. Sharma, A. *et al.* Brown fat determination and development from muscle precursor cells by novel action of bone morphogenetic protein 6. *PLoS One* **9**, (2014).

22. Min, S. Y. *et al.* Human ‘brite/beige’ adipocytes develop from capillary networks, and their implantation improves metabolic homeostasis in mice. *Nat. Med.* **22**, 312–318 (2016).

23. Kajimura, S., Spiegelman, B. M. & Seale, P. Brown and beige fat: Physiological roles beyond heat generation. *Cell Metab.* **22**, 546–559 (2015).

24. Iwen, K. A. *et al.* Cold-induced brown adipose tissue activity alters plasma fatty acids and improves glucose metabolism in men. *J. Clin. Endocrinol. Metab.* (2017) doi:10.1210/jc.2017-01250.

25. Wang, X. & Wahl, R. Responses of the insulin signaling pathways in the brown adipose tissue of rats following cold exposure. *PLoS One* **9**, (2014).

26. Kissig, M. *et al.* PRDM16 represses the type I interferon response in adipocytes to promote mitochondrial and thermogenic programing. *EMBO J.* **36**, 1528–1542 (2017).

27. Kong, X. *et al.* IRF4 is a key thermogenic transcriptional partner of PGC-1α. *Cell* **158**, 69–83 (2014).

28. Rahman, S. *et al.* Inducible brown adipose tissue, or beige fat, is anabolic for the skeleton. *Endocrinology* **154**, 2687–2701 (2013).

29. Yubero, P. *et al.* Dominant negative regulation by c-Jun of transcription of the uncoupling protein-1 gene through a proximal cAMP-regulatory element: a mechanism for repressing basal and norepinephrine-induced expression of the gene before brown adipocyte differentiation. *Mol. Endocrinol.* **12**, 1023–37 (1998).

30. Disilvestro, D. J. *et al.* Leptin production by encapsulated adipocytes increases brown fat, decreases resistin, and improves glucose intolerance in obese mice. *PLoS One* **11**, 1–18 (2016).

31. Beretta, E., Dhillon, H., Kalra, P. S. & Kalra, S. P. Central LIF gene therapy suppresses food intake, body weight, serum leptin and insulin for extended periods. *Peptides* **23**, 975–984 (2002).

32. Nam, M. *et al.* Mitochondrial retrograde signaling connects respiratory capacity to thermogenic gene expression. *Sci. Rep.* **7**, 2013 (2017).

33. Olsen, J. M. *et al.* β3-Adrenergically induced glucose uptake in brown adipose tissue is independent of UCP1 presence or activity: Mediation through the mTOR pathway. *Mol. Metab.* **6**, 611–619 (2017).

34. Camerino, C. *et al.* Nerve growth factor, brain-derived neurotrophic factor and osteocalcin gene relationship in energy regulation, bone homeostasis and reproductive organs analyzed by mrna quantitative evaluation and linear correlation analysis. *Front. Physiol.* **7**, 1–9 (2016).

35. Nakahara, K. *et al.* Involvement of endogenous neuromedin U and neuromedin S in thermoregulation. *Biochem. Biophys. Res. Commun.* **470**, 930–935 (2016).

36. Su, Y., Foppen, E., Fliers, E. & Kalsbeek, A. Effects of intracerebroventricular administration of neuropeptide Y on metabolic gene expression and energy metabolism in male rats. *Endocrinology* **157**, 3070–3085 (2016).

37. Nam, D. *et al.* Novel Function of Rev-erbα in Promoting Brown Adipogenesis. *Sci. Rep.* **5**, 11239 (2015).

38. Duteil, D. *et al.* Lsd1 Ablation Triggers Metabolic Reprogramming of Brown Adipose Tissue. *Cell Rep.* **17**, 1008–1021 (2016).

39. Kiskinis, E. *et al.* RIP140 Represses the “Brown-in-White” Adipocyte Program Including a Futile Cycle of Triacyclglycerol Breakdown and Synthesis. *Mol. Endocrinol.* **28**, 344–356 (2014).

40. Li, G., Zhang, Y., Wilsey, J. T. & Scarpace, P. J. Hypothalamic pro-opiomelanocortin gene delivery ameliorates obesity and glucose intolerance in aged rats. *Diabetologia* **48**, 2376–2385 (2005).

41. Chou, C. F. *et al.* KSRP ablation enhances brown fat gene program in white adipose tissue through reduced miR-150 expression. *Diabetes* **63**, 2949–2961 (2014).

42. Scheele, C. & Nielsen, S. Metabolic regulation and the anti-obesity perspectives of human brown fat. *Redox Biol.* **12**, 770–775 (2017).

43. Bargut, T. C. L., Aguila, M. B. & Mandarim-de-Lacerda, C. A. Brown adipose tissue: Updates in cellular and molecular biology. *Tissue Cell* **48**, 452–460 (2016).

44. Martínez De Morentin, P. B. *et al.* Nicotine induces negative energy balance through hypothalamic AMP-activated protein kinase. *Diabetes* **61**, 807–817 (2012).

45. Aguirre, L. *et al.* Effects of pterostilbene in brown adipose tissue from obese rats. *J. Physiol. Biochem.* 1–8 (2017) doi:10.1007/s13105-017-0556-2.

46. Bal, N. C. *et al.* Mild cold induced thermogenesis: Are BAT and skeletal muscle synergistic partners? *Biosci. Rep.* **37**, (2017).

47. Viengchareun, S., Zennaro, M. C., Tallec, L. P. Le & Lombes, M. Brown adipocytes are novel sites of expression and regulation of adiponectin and resistin. *FEBS Lett.* **532**, 345–350 (2002).

48. Ro, S.-H. *et al.* Sestrin2 inhibits uncoupling protein 1 expression through suppressing reactive oxygen species. *Proc. Natl. Acad. Sci.* **111**, 7849–7854 (2014).

49. Mur, C., Valverde, A. M., Kahn, C. R. & Benito, M. Increased Insulin Sensitivity in IGF-I Receptor–Deficient Brown Adipocytes. *Diabetes* **51**, 743–754 (2002).

50. Giralt, A. & Villarroya, F. SIRT3, a pivotal actor in mitochondrial functions: metabolism, cell death and aging. *Biochem. J.* **444**, 1–10 (2012).

51. Zhang, H. *et al.* Cross Talk between Insulin and Bone Morphogenetic Protein Signaling Systems in Brown Adipogenesis. *Mol. Cell. Biol.* **30**, 4224–4233 (2010).

52. Villena, J. A. *et al.* Mitochondrial biogenesis in brown adipose tissue is associated with differential expression of transcription regulatory factors. *Cell. Mol. Life Sci* **59**, 1934–1944 (2002).

53. Cawthorn, W. P. & Sethi, J. K. TNF-alpha and adipocyte biology. *FEBS Lett.* **582**, 117–131 (2008).

54. Golozoubova, V., Cannon, B. & Nedergaard, J. UCP1 is essential for adaptive adrenergic nonshivering thermogenesis. *Am. J. Physiol. Endocrinol. Metab.* **291**, E350–E357 (2006).

55. Caron, A. *et al.* Loss of UCP2 impairs cold-induced non-shivering thermogenesis by promoting a shift toward glucose utilization in brown adipose tissue. *Biochimie* **134**, 118–126 (2017).

56. Riley, C. L. *et al.* The complementary and divergent roles of uncoupling proteins 1 and 3 in thermoregulation. *J. Physiol.* **594**, 7455–7464 (2016).

57. Verdeguer, F. *et al.* Brown Adipose YY1 Deficiency Activates Expression of Secreted Proteins Linked to Energy Expenditure and Prevents Diet-Induced Obesity. *Mol. Cell. Biol.* **36**, MCB.00722-15 (2015).
